# Supplementary material for: Are carbon emissions trading and green financial instruments synergistic? -Comprehensive quantitative research based on content analysis
Source: PLoS One. 2024 Mar 7;19(3):e0298601. doi: 10.1371/journal.pone.0298601 (PMC10919601; doi:10.1371/journal.pone.0298601)
Supplement: S1 Data — (ZIP) [file pone.0298601.s001.zip › Data/地级市Z_F工作报告.zip!/░▓╗╒/╬▀║■╩╨/└┤╫╘╣1⁄2╓┌║┼í╛┬φ┐╦╩2╛▌═°í┐_2013.docx]

——2013年1月6日在芜湖市第十五届人民代表大会第一次会议上

市长 杨敬农

各位代表：

现在，我代表市人民政府，向大会报告本届政府过去五年的工作，对今后五年及今年工作提出建议，请各位代表审议，并请市政协委员和其他列席人员提出意见。

一、奋发图强，实现又好又快发展

过去的五年，是芜湖人民同心协力、奋发图强的五年，也是芜湖经济社会实现大发展、大跨越的五年。在中共芜湖市委的坚强领导下，全市上下深入贯彻党的十七大精神，认真落实中央和省委、省政府决策部署，紧紧抓住皖江城市带承接产业转移示范区、合芜蚌自主创新综合试验区建设、行政区划调整等重大战略机遇，积极应对严峻复杂的宏观环境，锐意改革，开拓创新，经济社会发展取得了新的重大成就。

五年来，综合实力迈上新台阶。主要经济指标总量跃居全省第二位，占全省比重均超过10%。地区生产总值由717亿元增加到1880亿元（预计数，下同），年均增长15.9%，是改革开放以来增长最快的时期。人均地区生产总值7820美元，是2007年的3倍。财政收入由116亿元增加到337.1亿元，年均增长23.7%。完成固定资产投资6274亿元，年均增长35%。社会消费品零售总额由208亿元增加到490亿元，年均增长18.7%。进出口总额由17亿美元增加到46.4亿美元，年均增长22.3%。金融机构本外币存款余额由667亿元增加到1876.3亿元；贷款余额由504亿元增加到1725.9亿元。建成了奇瑞整车改扩建、海螺日产1.2万吨水泥熟料、美的日电基地、新兴铸管三山基地、格力空调一期、瑞丰商品交易博览城、方特梦幻王国主题公园等一批重大项目。工业化率由48.6%上升到59.6%。规模工业企业由433家增加到1718家，新增产值超百亿元企业3家。汽车及零部件、材料、家用电器、电线电缆四个支柱产业不断壮大，产值占规模工业比重达69.2%。高新技术企业由2008年的145家增加到344家；高新技术产业增加值占地区生产总值比重由17.6%提高到28.2%；新增省级以上研发机构131个；申请专利4.1万件、授权专利2.3万件。新增中国驰名商标12件、国家级质检中心2个。芜湖高新区升格为国家高新技术产业开发区。服务业布局进一步优化，商业业态更加丰富，创建了金融、服务外包、中央商务区、总部经济4个省级现代服务业集聚区。船舶交易市场交易量位居全国第一。农业综合生产能力提高，粮食产量达134万吨，连续九年增产。建立省级以上现代农业示范区4个，省级以上农业产业化龙头企业达53家。

五年来，城乡建设展现新面貌。宁安城际铁路、合福高铁、芜申运河开工建设，芜雁高速公路建成通车。完成城市基础设施投资673亿元，市区面积由720平方公里扩大到1065平方公里，城市空间布局更加优化，城东新区建设全面展开。城镇化率由49.8%上升到58.3%，是改革开放以来城镇化推进速度最快的时期。建成城镇道路680公里、农村“村村通”水泥路3200公里，改造危桥170座。城市日供水能力由40万吨增加到77万吨。四县都新建了污水处理厂，全市污水日处理能力由10万吨增加到51万吨。在全省率先实现城乡生活垃圾处理全覆盖。发电装机容量由26.6万千瓦增加到396.8万千瓦，全社会年用电量由62.9亿千瓦时增加到127亿千瓦时。天然气年供应量由1.3亿立方米增加到2.7亿立方米。实施了保兴垾综合整治等一批绿化、美化、亮化和雨污分流工程，新增绿化面积1820万平方米。实施了45条道路架空杆线下地。新增公交车746辆、出租车954辆。整治了204个老旧小区。实施棚户区改造，建设安置房337万平方米，4.6万户居民受益。建设保障性住房12.6万套、617万平方米。我省单体投资规模最大的水利工程——青弋江分洪道开工建设。除险加固62座水库，建成了江南片万亩圩口防洪达标等水利项目，启动中小河流治理工程，解决了97万农村人口饮水安全问题。创建全国环境优美乡镇8个，南陵县成为国家级生态示范区。全面完成了省政府下达的节能减排任务。

五年来，改革开放实现新突破。医药卫生体制改革取得成效，在全国率先实施了医药分开改革，实行药品零差率销售，减轻群众药费负担3.8亿元。推进全国公立医院改革试点，成立三大医疗集团，医疗服务向区级医院和社区卫生机构延伸。国有资本运营能力显著增强，市级国有经营性资产由131亿元增加到807亿元。民营经济健康发展，新增私营企业1.4万户、个体工商户5.3万户，入围全省民营企业百强和出口创汇百强的企业数位居全省第一，非公有制经济比重达64.5%。农村集体土地确权发证和集体林权制度主体改革基本完成，流转耕地106万亩、林地16.8万亩。土地整治全面推进，新增耕地5.7万亩。南陵县成为全国农田水利建设投资和建管体制改革试点县。农村信用社全部改制成为合作银行或商业银行，组建村镇银行2家，引进全国性股份制银行5家，皖江金融租赁公司经国家批准设立，新增小额贷款公司62家、法人典当行14家，重新核准融资性担保机构38家。调整了市与区财税体制，增强区级财力，加强财政资金和建设资金统一调度管理，完成了事业单位资产资金清理集中工作。清理行政事业性收费，年减轻企业负担1.2亿元。率先实施事业单位分类改革，市属事业单位精简42.5%。坚持公开公平竞争原则，不断完善政府招标采购制度。省江北产业集中区管理体制调整为“省市共建、以市为主”，15平方公里起步区建设全面推进。芜湖承接产业转移集中示范园区获省政府批准。亳州芜湖现代产业园区建设全面启动。全市实际利用内资5250亿元、外资43亿美元，新增境外世界500强企业来芜投资项目18个。开展进出口业务的企业由220家增加到530家。港口货物吞吐量由4600万吨增加到8260万吨。奇瑞、海螺等企业实施“走出去”战略取得新进展。

五年来，民生水平得到新提升。投入207亿元，滚动实施48项民生工程。新增就业39万人；发放小额担保贷款20.5亿元，位居全省第一。覆盖城乡的社会保障体系基本建立，基本养老和基本医疗保险实现政策全覆盖。职工基本养老、医疗、失业、工伤、生育保险参保人数由125万人次提高到211万人次。企业退休人员月均养老金由868元增加到1470元，最低工资标准由每月540元提高到800元。在全省率先建立低保标准自然增长、临时救助等制度，城乡低保月人均补差从119元、35元分别提高到335元、138元。农村五保户集中供养率从不足20%上升到50%以上。迁址新建社会福利院和救助站，全市为汶川特大地震捐款近亿元，慈善事业健康发展。医疗卫生机构布局进一步优化、设施明显改善，新增病床近3000张。义务教育经费保障机制和中考招录制度不断完善，教育均衡发展成效显著。新建、改扩建中小学590所，完成了校舍安全工程和375所学校标准化建设。实施学前教育三年行动计划，新增公办幼儿园35所，扶持普惠性幼儿园146所。高中阶段毛入学率由62%上升到89.4%。建成国家级中等职业教育改革和发展示范学校2所。安徽工程大学机电学院独立办学，民办扬子职业技术学院建成招生。市科技馆、文化馆、图书馆建成使用，新建综合文化站74个、农家书屋911个，公共图书馆、文化馆（站）全部免费开放。新建改造标准化菜市场46处。承办了三届科普产品博览交易会、两届国际动漫创意产业交易会和央视中秋晚会。我市运动健儿在北京奥运会、广州亚运会等重大赛事中取得好成绩。国防动员、双拥优抚、兵役工作进一步加强。开展了第六次全国人口普查。档案、地方志、民族、宗教、气象、外事、侨务、对台、妇女儿童、老龄、残疾人等事业取得新进步。城乡居民人均收入分别由13234元、4712元增加到24550元、9760元，年均分别增长13.2%和15.7%。

五年来，政府建设取得新成效。自觉接受市人大及其常委会的法律监督、工作监督和市政协的民主监督，主动加强与各民主党派、工商联、各人民团体的联系，认真听取各界人士的意见和建议，办理人大议案和建议589件、政协提案1813件，回复网民咨询和市长信箱留言3.4万条。“五五”普法顺利完成，“六五”普法全面开展。完成了第七、第八届村委会和第六、第七届社区居委会换届选举，和谐社区建设扎实推进。制定市政府工作规则、重大行政决策程序规定，规范了决策行为。深入开展相对集中行政处罚权工作，行政执法行为不断规范。建成电子政务系统，市政府成为国家行政管理与公共服务标准化试点单位。推行建设项目“并联审批”、企业注册登记“一表通”、收费管理“一票制”服务，完善了政务服务网络。深入开展机关效能建设，加强廉政风险防控，开展工程建设领域突出问题、“小金库”等专项治理，对违纪违法人员坚决严肃查处。

过去五年里，在全市上下共同努力下，我市荣获多项国家级殊荣。蝉联全国科技进步先进市、全国文化体制改革先进地区、全国双拥模范城，成为国家知识产权示范城市、全国创业先进城市、全国流通领域现代物流示范城市、国家园林城市、全国可再生能源建筑应用示范城市、全国新能源示范城市、全国人民防空先进城市、全国无偿献血先进城市、全国人口和计划生育综合改革示范市、全国未成年人思想道德建设工作先进城市，两次荣获中国人居环境范例奖。我市被列入在中国特色社会主义旗帜指引下开拓成功发展之路的全国18个典型地区之一。

2012年是本届政府任期的最后一年。一年来，我们牢牢把握稳中求好、好中求快的总要求，扎实推进各项工作，保持了经济社会发展的良好势头。预计全市实现地区生产总值比上年增长13.8%，财政收入增长15.9%，全社会固定资产投资增长24%，社会消费品零售总额增长16.5%，进出口总额增长17%，城乡居民人均收入均增长14%以上，城镇新增就业8.5万人，城镇登记失业率3.5%，人口出生率9.35‰。

一年来，重点抓了以下几个方面工作：

一是全力以赴稳增长。加大对实体经济发展的支持力度，落实国家减、免、退税政策，兑付60.8亿元。减缴、缓缴社会保险费7.2亿元，发放稳岗补贴和促进就业补贴1.1亿元。出台了关于促进经济平稳较快发展的43条实施意见，召开了中小企业、金融等14个座谈会，开展政策对接，提振企业信心。强化重点项目服务，坚持市领导联系重大项目制度，奇瑞重工农业装备、双钱轮胎、镜湖万达广场等一批大项目建成。着力缓解中小企业融资难，组建了注册资金10亿元的民强担保集团，成立了市中小企业金融服务中心，金融机构新增贷款293亿元。市建投公司、经开区、高新区共发债53亿元，奇瑞发行10亿元私募债券，在全省率先发行中小企业集合债券、集合票据，全市直接融资197亿元。

二是持之以恒推创新。出台了企业股权和分红激励试点配套政策，支持企业创新。鑫龙电器成为国家创新型试点企业。新增3家国家级、27家省级研发机构和2家院士工作站，新增高新技术企业60家、高新技术产品180个、科技企业孵化器5.3万平方米。芜湖留学生创业园列入省部共建项目。新芜经济开发区成为省级高新技术产业开发区。全国特种电缆产业知名品牌示范区、国家特种电线电缆质检中心和3个省级质检中心获批建设。5家企业获首届市政府质量奖。培育培训各类人才2.9万人，引进和培育高端人才1720人、创新创业团队40个。

三是毫不动摇调结构。将汽车及装备制造确定为全市首位产业，实现增加值424亿元，增长23%。积极培育战略性新兴产业，实现增加值242亿元，增长35%。出台了关于进一步加快服务业发展若干政策的意见、建设现代服务业集聚区的实施意见。安徽国家数字出版基地芜湖园区获批，中国非物质文化博览园开工建设，中外运三山码头、服务外包产业园、文化创意产业园、南翔万商等项目建设加快。文化创意企业达207家。服务外包收入达2亿美元。游客接待量2200万人次，增长51.3%。举办了中国（芜湖）电线电缆博览会、第二届“刘开渠奖”国际雕塑大展等22项会展。实施了12万亩国家优质油菜生产基地、1.5万亩中央财政森林抚育补贴试点、1万亩蔬菜标准园项目建设，“陶辛青虾”、紫云英“弋江籽”成为国家农产品地理标志品牌。全力开展产业研究、企业服务和招商引智，全年实际利用内资1680亿元、外资13亿美元，均增长23.3%；新增规模以上工业企业294个、限额以上服务业单位369个。

四是城乡并重抓建设。投入196亿元，推进城市基础设施建设。修编了全市“十二五”规划和城市总体规划。出台了关于加快建设江北、推进跨江发展的26条意见。天门山路拓宽工程、花津桥、清水河公路桥等建成通车，205和318国道改造、中江大道南延线等开工建设。利民路水厂二期工程竣工。两座110千伏以上输变电工程建成运行。建成了公共自行车租赁系统，安排投放自行车1.2万辆。完成房地产投资362亿元，增长18%；商品房销售480万平方米，增长18%。新改建农村公路145公里，改造危桥40座、农村危房7405户。实施了无为万亩圩口防洪达标等一批水利工程。启动美好乡村建设，改造提升59个示范村。

五是多措并举优环境。启动国家生态园林城市创建工作，大阳垾湿地公园等13座城市公园正加快建设。实施森林增长工程，完成营造林8万亩。完成30个住宅小区雨污分流改造。出台了关于加强和创新城市管理工作的意见，开展了市容整治规范年、公共文明指数测评活动，推进网格化、信息化管理。实施节能项目72个，启动节能Ⅲ级预警，加强对64户高耗能企业的监管。以水泥厂低氮燃烧改造等为重点，实施减排项目78个。整顿关闭非煤矿山19家、小煤矿4家，全市煤矿企业全部关停。制定实施了机动车排气污染防治管理办法。通过土地整治增加1.3万亩耕地和6476亩城乡建设用地增减挂钩周转指标，盘活闲置和低效用地8500亩。竣工标准化厂房110万平方米。启动了繁昌循环经济产业园建设。我市成为全国第二批餐厨废弃物资源化利用和无害化处理试点城市。

六是坚持不懈促改革。稳步实施医药卫生体制改革，建立了大病医疗补充保险制度。营业税改征增值税试点顺利实施。制定了融资性担保机构、小额贷款公司、典当行经营资格年度检查及高级管理人员任职资格管理办法，加强征信系统建设，积极防范金融风险。开展了水利建管体制改革。推进了工矿废弃地复垦利用、国家级开发区存量建设用地二次开发利用等土地管理试点。启动镜湖区社区管理体制综合改革，减少管理层级，推行综合执法，强化便民服务。实施了“企业一套表”统计改革和人口管理信息化建设。

七是用心用情惠民生。政府性投入71亿元，完成了39项民生工程任务。推进创业富民，新增就业岗位9.8万个。城乡居民养老保险参保达171万人，45万人享受了养老待遇。城镇居民医疗保险参保达92.2万人，新型农村合作医疗参保达226.6万人。在全省率先启动了社会保障卡发放工作。出台了城乡居民最低生活保障实施细则，支付低保金4.2亿元，保障低保对象16万人。发放个人住房公积金贷款18亿元。保障性安居工程新开工6.2万套，竣工0.9万套。在全省率先推行全免费义务教育，实现了学生资助制度从学前教育到高等教育的全覆盖。完成中小学校舍安全工程6.3万平方米，改造农村小学教学点80个。在全省率先实现免费孕前优生健康检查全覆盖。出台了进一步做好农民工、留守儿童、空巢老人等特殊群体帮扶解困工作的意见，对80岁以上老人发放高龄津贴2600万元。推进机关联系基层、干部联系群众和大接访活动，加强社会矛盾纠纷调解。启动食品药品安全示范创建和量化分级管理，挂牌整改安全生产重大隐患9处，安全生产形势平稳向好。加强社会治安综合管理，社会大局和谐稳定。

回首过去五年攻坚克难、砥砺奋进的历程，积累的许多经验弥足珍贵，应当始终坚持，主要有：

必须坚持解放思想。弘扬崇尚创新、宽容失败、支持冒险、鼓励冒尖的创新文化，与时俱进，敢为人先。坚持改革开放，努力破除制约发展的思想障碍和体制桎梏，充分利用国际国内两个市场、两种资源，不断优化发展环境，让各类市场主体的活力竞相迸发，让一切创造财富的源泉充分涌流。

必须坚持产业强市。始终把招商引资作为经济工作的重中之重，把项目建设作为经济工作的生命线，坚持有所为、有所不为，培育一批具有核心竞争力的龙头企业，扶持一批成长性好的中小企业，以产业集群化、高端化支撑城市转型崛起。

必须坚持城乡统筹。树立以工促农、以城带乡理念，大力推进城乡一体化发展，以城乡规划全覆盖为引领，以城乡基础设施建设协调推进为突破，以城乡产业合理布局为重点，以公共服务均等化为核心，实现功能提升、服务完善、资源节约、环境友好，增强可持续发展能力。

必须坚持民生为要。时刻把群众的安危冷暖放在心上，做到问政于民、问需于民、问计于民，多为群众办好事、办实事，大力保障改善民生，维护社会公平正义，促进人的全面发展，让人民群众共享改革发展成果。

必须坚持服务至上。强化公仆意识和责任意识，经济发展围绕企业家特别是优秀企业家“转”，社会建设围绕老百姓特别是困难群众“转”，政府服务围绕基层工作特别是急事难事“转” ，落实“一线工作法”，帮助企业、群众、基层解决实际困难和问题。

各位代表！

奋斗充满艰辛，奉献铸就辉煌。过去五年的成就来之不易，这是中共芜湖市委统揽全局、坚强领导的结果，是人大代表充分行使职权、政协委员积极参政议政的结果，是全市人民奋力进取、各界人士同心同德、广大干部宵旰图治的结果。在此，我谨代表市人民政府，向全市各级人大代表、政协委员，向全市广大工人、农民、知识分子、干部、驻芜解放军指战员、武警官兵和政法干警，向各民主党派、工商联、各人民团体、无党派人士、离退休老同志和社会各界人士，向在我市创业的投资者、建设者，向关心支持芜湖发展的中央、省直各部门和海内外友好人士，表示崇高的敬意和衷心的感谢！

各位代表！

总结过去，我们充分认识到，前进道路上还面临很多挑战，工作中还存在许多不足，主要是：经济规模还不够大，产业结构还不够优，抗风险能力还不够强；产业转型升级步伐不快，战略性新兴产业发展未取得根本性突破，自主创新体制机制仍需完善；资源环境约束加剧，发展中不平衡、不协调、不可持续的问题依然突出，深化改革开放任务艰巨；城市布局和形态有待优化，功能和品位仍需提升，城乡发展差距较大；社会事业发展不平衡，公共服务均等化水平不够高，社会管理创新任务繁重；民生保障水平还比较低，城乡居民收入有待提高，部分群众生活还比较困难。一些干部领导科学发展能力需要进一步提高，有的地方发展环境需要进一步改善，政府服务效能需要进一步提升；一些部门消极腐败、奢侈浪费现象时有发生，一些领域道德失范、诚信缺失问题依然存在。

我们一定以对国家和人民高度负责的精神，采取更加有力的措施，切实解决这些问题，绝不辜负全市人民的期望和重托。

二、奋勇前进，率先建成小康社会

今后五年是我市全面实现小康社会宏伟蓝图的决定时期，也是加快推进现代化大城市建设的关键时期。综合分析国内外形势，虽然国际金融危机深层次影响远未消除，但世界经济缓慢复苏的趋势没有变，我国新型工业化、信息化、城镇化、农业现代化加速推进的形势没有变，我市厚积薄发、加速崛起的态势没有变。芜湖作为全省“双核”城市之一，区位交通、产业基础、生态环境、政策支持等比较优势明显，内生增长的动力更加强劲，对外合作的能力日益提升，我们完全可以发展得更好一些、更快一些。面向未来，我们要牢牢扭住经济建设这个中心，聚精会神搞建设，一心一意谋发展，沿着中国特色社会主义道路奋勇前进，在全省率先全面建成小康社会、率先基本实现现代化。

未来五年政府工作的基本思路是：全面贯彻党的十八大精神和省、市第九次党代会战略部署，坚持以科学发展为主题，以全面转型、率先崛起、富民强市为主线，以率先建成小康社会为目标，大力实施“创新驱动、产业强市、统筹城乡、开放合作、和谐发展”五大战略，加快推进产业高端化、城市现代化、环境生态化，着力打造宜居宜业宜游的现代化大城市，努力建设创新、优美、和谐、幸福新芜湖。

未来五年经济社会发展的主要目标是：

——经济更加发达。转变经济发展方式取得重大进展，发展质量和效益明显提高，产业整体素质大幅提升，自主创新能力显著增强，城市综合实力实现新的跨越，把芜湖建设成为长江流域具有较强创新能力的经济文化强市。地区生产总值年均增长13%以上，财政收入同步增长。

——环境更加优美。生态文明建设深入推进，城乡生态环境持续改善，山水人文特色彰显，城市综合配套功能日臻完善，城市品位和现代化水平大幅提升，把芜湖建设成为大气、秀美、时尚、欢乐的国家生态园林城市。2017年城镇化率达到68%、森林覆盖率达到24%以上。

——社会更加和谐。经济社会全面协调可持续发展，社会管理更为科学，文明程度大幅提高，城乡居民对城市的归属感、安全感和自豪感明显增强，把芜湖建设成为民主法治、公平正义、诚信友爱、安定有序的文明之城、和谐之城。

——人民更加幸福。就业更为充分，民生保障更为完善，全民受教育程度普遍提高，居民财产性收入普遍增加，人民群众物质生活殷实宽裕、精神文化生活健康丰富，把芜湖建设成为文化教育发达、健康水平较高、生活品质优良的幸福之城。2017年城乡居民收入比2012年翻一番。

在未来五年的工作中，要把握好以下几个方面：

第一，推进转型发展，着力提升产业层次。坚持先进制造业与现代服务业“双轮驱动”，促进产业结构从二产为主向二、三产业并重转变，发展动力从以投资拉动为主向以消费拉动、创新驱动为主转变。把汽车及装备制造业作为全市首位产业长期培育，壮大智能装备制造业，大力发展光电、新能源汽车、智能家电、节能环保装备、新材料、生物医药六大新兴产业，打造具有较强国际竞争力、全国重要的先进制造业基地，培育一批销售收入超千亿元产业。引导企业走现代管理之路，深入实施标准化和品牌战略，培育一批具有核心竞争力的龙头企业，支持奇瑞、海螺、新兴铸管早日壮大成为世界500强企业。大力发展现代服务业，推进服务业规模扩张、结构优化和竞争力提升。加快发展金融、现代物流和服务外包，打造皖江金融中心、皖江航运中心、全国重要的现代物流产业中心、国家级服务外包基地。建设文化旅游强市，促进文化创意和科技、旅游融合，新建一批特色主题公园，推进皖南国际文化旅游示范区建设，使文化旅游产业发展成为支柱产业，把芜湖打造成为长三角文化创意产业集聚区、全国有重要影响力的旅游目的地。以土地流转、规模经营、产业化发展为抓手，以高效生态、特色精品为重点，建设都市型现代农业示范区。

第二，推进拥江发展，着力统筹城乡建设。坚持高起点规划、组团式发展，举全市之力推进拥江发展，促进区域均衡、城乡一体、功能互补、联动融合、共同繁荣。全面提升主城区，建设江南城区、龙湖新城、江北新城三大组团；推动无城、湾沚、繁阳、籍山4 座县城发展成为中等城市；在省级以上开发区周边规划建设 12 个产业新城；打造高沟、石涧、襄安、许镇、弋江、荻港、六郎7个新市镇，建设11个中心镇，使之成为县域特色产业和城镇人口的重要集聚地、带动乡村发展的服务基地；实施1000个村庄整治工程，建设美好乡村。加快构建综合交通体系，建成宁安城际铁路、合福高铁、皖赣新建双线、芜湖长江公路二桥、商合杭铁路芜湖长江公铁大桥、铜南宣高速公路、芜黄高速公路、北沿江高速公路无为段、芜申运河，推进泰山路长江大桥、城南过江隧道等过江通道建设，实现所有县城环城高速公路、客运铁路全覆盖。完成火车站、长途汽车客运站改扩建，全力推进民用机场建设。芜湖港跨入“亿吨大港”行列。建成青弋江分洪道工程，实施裕溪河及西河等重要支流治理。实现县县通天然气。完成中心城区路网畅通工程和棚户区改造。完善市容、市政、绿化、河道养护等领域管理体制，健全运行高效、服务完善、监管有力的现代城市管理模式。推进绿色发展、循环发展、低碳发展，强化资源节约、自然生态系统和环境保护。

第三，推进创新发展，着力深化改革开放。进一步转变政府职能，构建充满活力、富有效率、有利于科学发展的行政管理体制机制。深化国有资产管理制度改革，推动国有资本更多投向事关发展大局的重要行业和关键领域，促进国有资产保值增值。支持非公有制经济发展，完善“小巨人”企业培育计划，创新小微企业扶持方式，推动一批创新型中小企业爆发式增长。加快发展市场中介组织，健全和活跃土地、资本、技术、产权、人力资源、管理等要素市场。深化金融改革，引进外资银行，培育地方法人银行和民营金融机构，提高金融监管水平。充分利用资本市场融资，加快形成上市公司芜湖板块。加快改革户籍制度，有序推进农业转移人口市民化，努力实现城镇基本公共服务常住人口全覆盖。把创新驱动作为城市发展的首要战略，把提高自主创新能力作为提升城市核心竞争力的中心环节，完善鼓励研发投入、科技成果转化、创新平台建设、人才引进培育等政策措施，完善促进创新的体制机制，加快新技术研发应用，培育一批拥有自主品牌和核心技术的高新技术企业。充分利用皖江城市带承接产业转移示范区的先行先试权，全面提高开放型经济水平。以首位产业、战略性新兴产业和现代服务业为重点，引进一批投资规模大、技术含量高、带动能力强的重大项目。在更高层次上推进开发区建设，打造一批产值超千亿元开发区。加快“走出去”步伐，增强企业国际化经营能力。

第四，推进和谐发展，着力加强社会管理。健全党委领导、政府负责、社会协同、公众参与、法治保障的社会管理体制。把立德树人作为教育的根本任务，以教育信息化引领教育现代化，实现各类教育协调发展，决不让一个孩子因家庭贫困而失学，努力办好人民满意的教育。实施更加积极的就业政策，完善就业援助、技能培训和创业帮扶机制，鼓励创业，以创业带动就业，推动实现更高质量的就业。落实收入分配制度改革政策，千方百计增加城乡居民收入。坚持全覆盖、多层次、可持续方针，完善城乡社会保障体系。促进社会保险、社会救助、社会福利、慈善事业相互衔接，提高社会保障水平。切实保障老年人、残疾人和妇女儿童权益。深化医药卫生体制改革，完善公共卫生、医疗服务、药品供应体制，健全大病保障和救助机制，加强食品药品安全监管，提高人民健康水平。坚持计划生育的基本国策，提高出生人口素质，完善人口信息化管理。进一步扩大法律援助覆盖面。积极创新城市基层治理模式，增强城乡社区服务功能。巩固文明创建成果，争创全国文明城市。

各位代表！

率先全面建成惠及全市人民的小康社会，是贯彻落实党的十八大精神的具体行动，是全市人民共同的期待、共同的任务。我们一定要在中共芜湖市委的坚强领导下，以强烈的历史责任感和使命感，万众一心，团结奋斗，力争早日实现这一宏伟目标！

三、奋力作为，加速富民强市步伐

2013年是全面贯彻党的十八大精神的开局之年，也是实施“十二五”规划承前启后的关键一年，做好今年工作意义重大。综合各方面考虑，今年经济社会发展的主要预期目标初步安排是：地区生产总值比上年增长13%，财政收入与经济保持同步增长，全社会固定资产投资增长20%，社会消费品零售总额增长15%，进出口总额增长15%，城乡居民人均收入均增长15%左右，城镇新增就业7万人，节能减排完成省政府下达目标。

今年宏观经济形势总体向好，但仍然错综复杂，存在诸多不确定因素。我们清醒地认识到，内外需求不旺、部分企业产值增速下降、工业产品价格下跌、节能减排调控趋紧等因素对经济影响不断加深，去年我市地区生产总值、工业销售收入增幅尽管高于全国、全省平均水平，但未能实现更好预期。新一届政府要完成今年目标，压力很大，任务艰巨，必须开拓创新、苦干实干，必须只争朝夕、奋力作为，尤其要突出抓好以下七个方面工作：

（一）大力推动产业结构优化升级

聚焦突破首位产业，加快培育战略性新兴产业。建立健全首位产业统计、监测制度和考核激励办法，强化“一企一策”服务，推动优质生产要素向重点企业集中。加快建设奇瑞重工装备制造、海创节能装备制造等一批重大项目。发挥战略性新兴产业专项资金引导作用，申报设立装备制造、节能环保新兴产业创业投资基金，推进东旭液晶玻璃基板、信义三期、长信科技等新兴产业项目建设。推动信息化与工业化深度融合，促进优势传统产业和战略性新兴产业融合发展。

大力发展服务业。推进省级现代服务业集聚区建设，抓好镜湖区省级服务业综合改革试点工作。建成芜湖金融服务中心。加快构建区域股权投资基金中心，鼓励支持大型、优质股权投资企业进驻芜湖。推进企业上市，确保2家以上企业报会。加快推进安得物流综合物流园、中江物流基地、三山和江北公用码头及仓储等物流项目建设。充分发挥服务外包产业园等平台作用，吸引一批服务外包企业入驻。编制旅游发展总体规划，推动旅游项目建设，积极争创5A级旅游景区、三星级以上饭店和省级旅游餐馆、星级农家乐示范点、最佳旅游乡镇。加快南翔万商、万达广场二期等重点商贸项目建设。

积极发展现代农业。落实强农惠农富农政策，提升农业综合生产能力。推进种植业基地建设，深入实施畜牧业升级计划、水产跨越工程，加快省级现代农业示范区建设。推进安徽农机创新示范芜湖先行区建设。加快长江中下游地区（芜湖）农产品物联网工程等项目建设。以农业提质增效为基础，深入推进农业产业化、标准化。

（二）加快转变经济发展方式

扎实推进科技创新。积极开展企业股权和分红激励试点。推进国家科技和金融结合试点区工作。实施科技创新引领百家示范标杆企业培育计划，推进创新型产业集群试点。积极举办产学研合作活动。新增高新技术企业30家、创新型企业10家、高新技术产品130个，专利申请量1.3万件、授权量0.5万件。新增科技企业孵化器5万平方米，建成江北科技孵化中心和工业孵化器基地。新增省级以上工程技术研究中心和重点实验室8家，国家、省级重点科技计划项目90个。

推动节能减排和资源节约集约利用。加强节能监测和调控，创建信息化节能降耗示范企业。推进水泥熟料生产线低氮燃烧改造及脱硝工作，建成2台火电机组脱硝设施。机动车环保检验合格标志发放率达到90%以上。推进6个污水处理项目和餐厨废弃物处理项目建设。开展PM2.5（细颗粒物）监测工作。加强非煤矿山探矿权、开采权的整顿和管理，推进烟花爆竹生产企业有序退出。严格执行建设项目环境影响评价和环境准入制度。落实耕地保护制度，盘活存量土地。加大保障性住房、棚户区改造住房、中小套型普通商品住房“三类用地”供应力度。鼓励建设标准化厂房。

（三）全力推进城乡一体化进程

安排城市基础设施建设投资193亿元。开工建设芜湖长江公路二桥、铜南宣高速公路、新火车站、江北和谐大道、321省道改造、无为通江大道改造等项目。完成芜申运河东段、205国道示范改造、206省道沈巷段等工程项目建设。建成江北产业集中区起步区路网、长江南路、峨山路东延立交桥、沿河路防洪墙改造及道路扩宽工程，启动中江桥、弋江桥、荆山桥改造。建设3条城市道路公交专用道，新增268辆公交车，加强校车安全运营管理。加快推进旧城区改造。加强江北城区水系、保兴垾上游及支流、板城垾等水系整治。深入实施城市绿化、美化、亮化工程，加快公园建设，规划建设城市绿道系统。新增森林16.8万亩。完善网格化城市管理，落实考评和奖惩制度。扎实开展美好乡村建设，结合土地整治、农村危房改造和农村清洁工程，整合各类资源集中投入，建设85个中心村。开展农村沟渠湖塘清淤疏浚，完成22座小型水库除险加固和农村安全饮水、中小河流治理、无为万亩圩口防洪达标等工程年度建设任务。

（四）进一步深化改革开放

加强国有资产和投资管理。进一步深化事业单位分类改革，全面实施事业单位绩效工资改革。深入实施营业税改征增值税试点工作，深化税源专业化管理改革。贯彻实施城乡居民养老保险新政策，推进城乡养老保险制度衔接。继续深化医药卫生体制改革，重点抓好县级公立医院综合改革，完善公立医院管理体制机制，积极扶持社会资本办医。推动医保支付方式改革，开展全科医师执业方式和服务模式改革全国试点。推进城镇职工医疗保险和城镇居民基本医疗保险市级统筹。做好城乡住户统计调查一体化改革工作。推进旅游综合改革和投融资改革试点。加快推进省级以上开发区转型发展、优化升级，高标准建设省江北产业集中区，加快建设无为城东工业园。围绕首位产业、新兴产业、现代服务业龙头企业和产业链核心环节，开展精准招商。用足用活招才引智政策，吸引人才集聚。加快三山港外向型口岸建设，积极扩大对外贸易。加强城市之间的合作交流，支持亳州芜湖现代产业园区建设。

（五）着力改善民生和创新社会管理

安排政府性投入78亿元，实施40项民生工程。

加强就业和社会保障工作。推进校企合作，积极开发高校毕业生服务基层公益岗位。加强创业孵化载体建设，加大创业政策扶持力度，强力推进创业富民。实行社会保障全覆盖，建立商业保险机构承办大病医疗补充保险机制。加强社会保障信息化建设，推进金保工程二期建设。提高城乡居民最低生活保障水平，月人均补差标准比上年增长10%以上。健全特殊群体帮扶解困机制。新增农村五保供养床位800张，改扩建6家社会福利中心，建设20个居家养老服务站和社区日间照料中心。探索建立医疗护理型养老服务机构。加强保障房建设、管理和分配工作，竣工保障房2万套，扩大住房公积金制度覆盖面，让更多居民享受住房保障。

大力发展社会事业。全面完成学前教育三年行动计划。开展义务教育发展基本均衡县达标验收工作，完成农村小学优化布局改造工程。在中小学开展国学经典教育。推进职教园区建设，建设一批名师工作室。支持在芜高校发展。争创省级以上社区教育实验区、示范街道。完成城东新区医院一期工程建设，新建康复医院、市传染病医院新院区。深化以餐饮企业为重点的食品药品安全监管，建立网上公示制度。全面实施诚信计生，促进人口计生工作转型发展。完成城市气象监测预警中心、大阳垾标准气象观测站建设。深入开展“六五”普法和律师进社区工作。开展第三次全国经济普查。做好民族、宗教、外事、侨务等工作，发展档案、地方志、人防、防灾减灾等各项事业。

加强和创新社会管理。充分发挥人民团体作用，引导社会组织健康有序发展。适时推广镜湖区社区管理体制改革试点经验，打造15个精品社区。推进“智慧芜湖”建设，建成政务云计算中心、市级共享容灾备份中心，争取社区信息化国家试点。建成覆盖生产、流通、消费等各个环节的价格监测体系。支持驻芜部队建设，做好国防教育、优抚安置和双拥共建工作，增强全民国防意识。进一步完善信访制度，构建大信访工作格局，畅通和规范群众诉求表达、利益协调、权益保障渠道。强化公共安全体系和企业安全生产基础建设。推行居住证制度。完善立体化社会治安防控体系，建设互联网警务平台，排查化解矛盾纠纷，强化司法保障，依法防范和惩治违法犯罪活动。

（六）积极发展文化事业和文化产业

坚持用社会主义核心价值体系引领社会思潮、凝聚社会共识。加强社会公德、职业道德、家庭美德、个人品德教育，强化政务诚信、商务诚信、社会诚信建设。深入开展群众性精神文明创建活动，广泛开展志愿服务。深化文化体制改革，推进国有文化企业股份制改造，扶持民营文艺表演团体发展。推进芜湖古城等特色品牌项目建设。扶持文化产业发展，申报建设文化科技产业示范区、国家公共文化服务体系示范区，推进华强文化科技产业园、中国非物质文化博览园等重大项目建设。办好第四届中国国际动漫创意产业交易会、第三届“刘开渠奖”国际雕塑大展。建成市博物馆、规划馆和镜湖区图书馆、文化馆新馆，加快繁昌县、无为县图书馆新馆建设进度，推动公共文化服务体系向基层延伸。繁荣地方文艺创作，开展群众性文体活动和全民阅读活动。广泛开展主题科普活动，提高公众科学素养。加快奥园二期等体育设施建设，办好第七届中国体育旅游博览会、市第十二届运动会。

（七）努力建设人民满意的服务型政府

坚持依法行政，完善市政府工作规则，严格按照法定权限和程序行使权力、履行职责，办好人大议案和政协提案，健全行政执法责任制，推进依法行政示范单位创建工作。坚持为民行政，认真贯彻中央和省委关于改进工作作风的各项规定，坚持厉行节约、勤俭办一切事业，深入开展机关联系基层、干部联系群众活动，充分利用市民心声、市长热线等平台加强政府与市民的沟通交流。坚持实干富民、实干强市，着力加强学习和调查研究，着力增强解决实际问题的能力，着力整治庸懒散奢的不良风气。开展万人千企评议政风行风、民主评议百名科长等活动，强化监督考核机制。完善新闻发言人制度，加大政府信息公开力度，推进“阳光村务工程”建设，推动政务公开。坚持高效行政，推进行政管理与公共服务标准化建设试点，深化行政审批制度改革，简政放权，提高效率。坚持廉洁行政，全面推进惩治和预防腐败体系建设，严格规范权力行使，着力解决发生在群众身边的腐败问题，努力做到干部清正、政府清廉、政治清明。

各位代表！

芜湖的明天无限美好！我们的责任重大、使命光荣。让我们在中共芜湖市委的领导下，凝聚全市人民的智慧和力量，真抓实干，励精图治，倍道兼程，勇往直前，共同创造芜湖人民更加幸福、更加美好的未来！
